# Supplementary material for: Transcriptomic Analysis of the Porcine Gut in Response to Heat Stress and Dietary Soluble Fiber from Beet Pulp
Source: Genes (Basel). 2022 Aug 16;13(8):1456. doi: 10.3390/genes13081456 (PMC9408315; doi:10.3390/genes13081456)
Supplement: Supplementary file 1 [file genes-13-01456-s001.zip › genes-1830458-supplementary.pdf]

Table S1. Ingredients and nutrient composition in the experimental diets

Ingredients (%)	Basal diet	Basal diet + BP
Corn	70.93	66.68
Wheat	5.00	5.00
Oil	1.71	2.03
SBM (44%)	19.38	19.42
Sugar Beet Pulp	0.00	4.00
DL-Methionine (98%)	0.03	0.04
L-Lysine (78.8%)	0.22	0.20
Threonine (99%)	0.03	0.02
Limestone	0.72	0.62
DCP	1.22	1.23
Salt	0.20	0.20
Choline	0.05	0.05
Mineral premix[1]	0.15	0.15
Vitamin premix[2]	0.15	0.15
$NaCO_3$	0.16	0.16
Phytase	0.05	0.05
Total	100.0	100.0
**Nutrients**		
Metabolizable Energy (kcal/kg)	3,300	3,300
Crude Protein (%)	15.20	15.20
Calcium (%)	0.62	0.62
Total Phosphorus (%)	0.30	0.30
Lysine (%)	0.90	0.90
Methionine + Cysteine (%)	0.51	0.51
Threonine (%)	0.56	0.56
Tryptophane (%)	0.17	0.17

[1]Supplied per kilogram diet: 62.1 mg Fe; 4.1 mg Cu; 59 mg Zn; 2.1 mg Mn; 0.19 mg Se; and 0.14 mg I.

[2]Supplied per kilogram diet: 1,400 IU vitamin A; 160 IU vitamin D3; 12 IU vitamin E; 0.51 mg vitamin K3; 1.1 mg thiamine; 2.7 riboflavin; 9 mg pantothenic acid; 35 mg niacin; 1.1 mg pyridoxine; 0.07 mg biotin; 0.4 mg folic acid; 10 µg vitamin B12; and 350 mg choline.

Table S2. Genes differentially expressed in the porcine gut (HS vs. NT)

Gene Symbol	Gene Description	GenBank	p-Value
**Up-regulated genes**			
GSTA1	Glutathione S-transferase alpha 1	NM_214389.2	<0.001
HSPB6	Heat shock protein family B (small) member 6	XM_003127059.4	<0.001
PPP1R14A	Protein phosphatase 1 regulatory inhibitor subunit 14A	XM_021093577.1	<0.001
CKM	Creatine kinase, M-type	XM_021093542.1	<0.001
SYNM	Synemin	XM_021098348.1	<0.001
NEXM	Nexilin F-actin binding protein	XM_021096442.1	0.049
TAGLN	Transgelin	XM_005667371.3	0.001
C4BPA	Complement component 4 binding protein, alpha	XM_013979647.2	<0.001
CSRP1	Cysteine and glycine rich protein 1	XM_003357676.4	<0.001
TPM2	Tropomyosin 2 (beta)	XM_021080975.1	<0.001
MYLK	Myosin light chain kinase	XM_021070234.1	<0.001
SH3BGR	SH3 domain binding glutamate rich protein	NM_001244236.1	<0.001
PCP4	Purkinje cell protein 4	XM_021071000.1	<0.001
PDLIM3	PDZ and LIM domain 3	XM_005671717.3	0.003
DES	Desmin	XM_021074667.1	<0.001
MYL9	Myosin light chain 9	NM_001244472.1	<0.001
PYGM	Glycogen phosphorylase, muscle associated	XM_003122588.5	<0.001
CNN1	Calponin 1	NM_213878.1	<0.001
PDLIM7	PDZ and LIM domain 7	XM_021084376.1	<0.001
ASPN	Asporin	NM_001243889.1	0.035
ACTG2	Actin gamma 2, smooth muscle	XM_021087371.1	<0.001
FABP4	Fatty acid binding protein 4	NM_001002817.1	<0.001
S100A2	S100 calcium binding protein A2	XM_001929556.5	<0.001
TPM1	Tropomyosin 1 (alpha)	XM_005659518.3	<0.001

MGP	Matrix Gla protein	NM_214116.1	<0.001
MFAP5	Microfibril associated protein 5	XM_021090989.1	<0.001
**Down-regulated genes**			
MT1A	Metallothionein 1A	NM_001001266.2	<0.001
GPT2	Glutamic-pyruvic transaminase 2	XM_003126995.4	<0.001
TSPAN1	Tetraspanin 1	XM_005665450.3	<0.001
GZMB	Granzyme B	NM_001143710.1	<0.001
CD3E	CD3 epsilon subunit of T-cell receptor complex	NM_214227.1	<0.001
DDC	Dopa decarboxylase	XM_021063927.1	<0.001
ACE	Angiotensin I converting enzyme (peptidyl-dipeptidase A) 1	NM_001033015.3	<0.001
CCL4	C-C motif chemokine ligand 4	NM_213779.1	0.022
CCL5	C-C motif chemokine ligand 5	NM_001129946.1	<0.001
ITGAE	Integrin subunit alpha E	XM_021067683.1	<0.001
PMP22	Peripheral myelin protein 22	XM_021067896.1	<0.001
MX1	MX dynamin like GTPase 1	NM_214061.2	<0.001
DDX60	DExD/H-box helicase 60	XM_021072214.1	<0.001
C14H10orf99	Chromosome 14 C10orf99 homolog	NM_001243901.1	<0.001
GZMA	Granzyme A (granzyme 1, cytotoxic T-lymphocyte-associated serine esterase 3)	NM_001143709.1	<0.001
PCK1	Phosphoenolpyruvate carboxykinase 1	NM_001123158.1	<0.001
SLC5A12	Solute carrier family 5 member 12	XM_003122908.4	<0.001
CDHR2	Cadherin related family member 2	XM_013994874.2	<0.001
GNLY	Granulysin	NM_001278755.1	<0.001
PLB1	Phospholipase B1	XM_021087673.1	<0.001
APOB	Apolipoprotein B	NM_001375388.1	<0.001
GCNT3	Glucosaminyl (N-acetyl) transferase 3, mucin type	XM_021094516.1	<0.001
CLCA4	calcium-activated chloride channel regulator 4	XM_001926978.5	0.027

Table S3. Genes differentially expressed in the porcine gut (HS+BP vs. NT)

Gene Symbol	Gene Description	GenBank	p-Value
**Up-regulated genes**			
HSPB6	Heat shock protein family B (small) member 6	XM_003127059.4	<0.001
PPP1R14A	Protein phosphatase 1 regulatory inhibitor subunit 14A	XM_021093577.1	<0.001
CKM	Creatine kinase, M-type	XM_021093542.1	<0.001
DMPK	DM1 protein kinase	XM_021094591.1	<0.001
SYNM	Synemin	XM_021098348.1	<0.001
NEXM	Nexilin F-actin binding protein	XM_021096442.1	0.031
HSP70.2	Heat shock protein 70.2	NM_213766.1	0.045
GSTA1	Glutathione S-transferase alpha 1	NM_214389.2	<0.001
CFL2	Cofilin 2	NM_001025215.1	<0.001
SPARCL1			0.003
HBB	Hemoglobin, beta	NM_001144841.1	<0.001
CRYAB	Crystallin alpha B	XM_021062778.1	0.001
TAGLN	Transgelin	XM_005667371.3	0.001
C4BPA	Complement component 4 binding protein, alpha	XM_013979647.2	<0.001
IL33	Interleukin 33	NM_001285978.1	<0.001
CSRP1	Cysteine and glycine rich protein 1	XM_003357676.4	<0.001
TPM2	Tropomyosin 2 (beta)	XM_021080975.1	<0.001
MYLK	Myosin light chain kinase	XM_021070234.1	<0.001
SH3BGR	SH3 domain binding glutamate rich protein	NM_001244236.1	<0.001
PCP4	Purkinje cell protein 4	XM_021071000.1	<0.001
MSMB	Microseminoprotein beta	NM_213852.1	<0.001
PDLIM3	PDZ and LIM domain 3	XM_005671717.3	0.002
DES	Desmin	XM_021074667.1	<0.001

MYL9	Myosin light chain 9	NM_001244472.1	<0.001
CALD1	Caldesmon 1	XM_021079167.1	<0.001
PYGM	Glycogen phosphorylase, muscle associated	XM_003122588.5	<0.001
PLN	Phospholamban	NM_214213.1	<0.001
CNN1	Calponin 1	NM_213878.1	<0.001
PDLIM7	PDZ and LIM domain 7	XM_021084376.1	<0.001
MYH11	Myosin heavy chain 11	XM_021086165.1	0.002
OGN	Osteoglycin	NM_001315728.1	<0.001
HSPA6	Heat shock protein family A (Hsp70) member 6	NM_001123127.1	0.001
ACTG2	Actin gamma 2, smooth muscle	XM_021087371.1	<0.001
FABP4	Fatty acid binding protein 4	NM_001002817.1	<0.001
S100A2	S100 calcium binding protein A2	XM_001929556.5	<0.001
TPM1	Tropomyosin 1 (alpha)	XM_005659518.3	<0.001
LYZ	Lysozyme	NM_214392.2	0.001
MGP	matrix Gla protein	NM_214116.1	<0.001
**Down-regulated genes**			
GPT2	Glutamic-pyruvic transaminase 2	XM_003126995.4	<0.001
TSPAN1	Tetraspanin 1	XM_005665450.3	<0.001
SFTA2	surfactant associated 2	XM_003128304.3	<0.001
GZMB	Granzyme B	NM_001143710.1	<0.001
FOS	Fos proto-oncogene, AP-1 transcription factor subunit	NM_001123113.1	<0.001
DDC	Dopa decarboxylase	XM_021063927.1	<0.001
CCL4	C-C motif chemokine ligand 4	NM_213779.1	0.012
ITGAE	Integrin subunit alpha E	XM_021067683.1	<0.001
PMP22	Peripheral myelin protein 22	XM_021067896.1	<0.001
MX1	MX dynamin like GTPase 1	NM_214061.2	<0.001
C14H10orf99	Chromosome 14 C10orf99 homolog	NM_001243901.1	<0.001

GNLY	Granulysin	NM_001278755.1	<0.001
PLB1	Phospholipase B1	XM_021087673.1	<0.001
ST3GAL1	ST3 beta-galactoside alpha-2,3-sialyltransferase 1	XM_005662836.3	<0.001

Table S4. Genes differentially expressed in the porcine gut (HS+BP vs. HS)

Gene Symbol	Gene Description	GenBank	p-Value
**Up-regulated genes**			
CYP2J34	Cytochrome P450 family 2 subfamily J member 34	NM_001244633.1	<0.001
HBB	Hemoglobin, beta	NM_001144841.1	<0.001
HSPA6	Heat shock protein family A (Hsp70) member 6	NM_001123127.1	<0.001
S100A2	S100 calcium binding protein A2	XM_001929556.5	<0.001
GCNT3	Glucosaminyl (N-acetyl) transferase 3, mucin type	XM_021094516.1	<0.001
LYZ	Lysozyme	NM_214392.2	0.003
**Down-regulated genes**			
SCGB1A1	Secretoglobin family 1A member 1	XM_003353832.3	<0.001
LOC396781	IgG heavy chain	NM_213828.1	0.002
SAA3	Serum amyloid A-3 protein	XM_013994502.2	<0.001
ST3GAL1	ST3 beta-galactoside alpha-2,3-sialyltransferase 1	XM_005662836.3	<0.001
