# Supplementary figures and images for: Transcriptomic Analysis of the Porcine Gut in Response to Heat Stress and Dietary Soluble Fiber from Beet Pulp
Source: Genes (Basel). 2022 Aug 16;13(8):1456. doi: 10.3390/genes13081456 (PMC9408315; doi:10.3390/genes13081456)

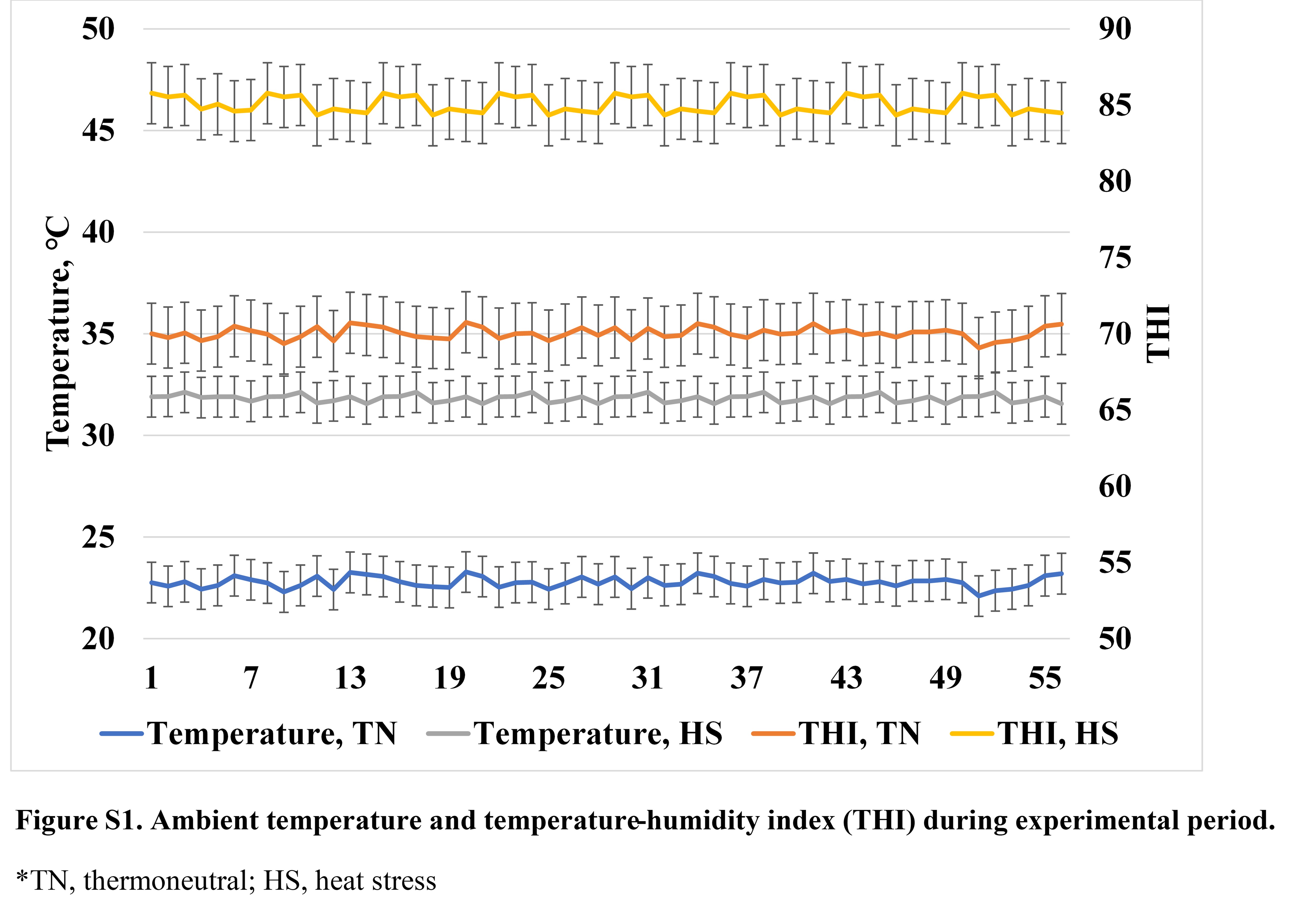

Supplement: Supplementary file 1 [file genes-13-01456-s001.zip › Supplementary_Figure S1.jpg]
